# Muscle Synergy Constraints Do Not Improve Estimates of Muscle Activity From Static Optimization During Gait for Unimpaired Children or Children With Cerebral Palsy

**DOI:** 10.3389/fnbot.2019.00102

**Published:** 2019-12-17

**Authors:** Benjamin R. Shuman, Marije Goudriaan, Kaat Desloovere, Michael H. Schwartz, Katherine M. Steele

**Affiliations:** ^1^Department of Mechanical Engineering, University of Washington, Seattle, WA, United States; ^2^Department of Human Movement Sciences, Faculty of Behavioural and Movement Sciences, Vrije Universiteit Amsterdam, Amsterdam, Netherlands; ^3^Department of Rehabilitation Sciences, KU Leuven, Leuven, Belgium; ^4^Clinical Motion Analysis Laboratory, University Hospitals Leuven (Pellenberg), Lubbeek, Belgium; ^5^James R. Gage Center for Gait and Motion Analysis, Gillette Children’s Specialty Healthcare, Saint Paul, MN, United States; ^6^Orthopaedic Surgery, Department of Biomedical Engineering, University of Minnesota, Minneapolis, MN, United States

**Keywords:** electromyography, muscle synergies, musculoskeletal modeling, cerebral palsy, static optimization

## Abstract

Neuromusculoskeletal simulation provides a promising platform to inform the design of assistive devices or inform rehabilitation. For these applications, a simulation must be able to accurately represent the person of interest, such as an individual with a neurologic injury. If a simulation fails to predict how an individual recruits and coordinates their muscles during movement, it will have limited utility for informing design or rehabilitation. While inverse dynamic simulations have previously been used to evaluate anticipated responses from interventions, like orthopedic surgery or orthoses, they frequently struggle to accurately estimate muscle activations, even for tasks like walking. The simulated muscle activity often fails to represent experimentally measured muscle activity from electromyographic (EMG) recordings. Research has theorized that the nervous system may simplify the range of possible activations used during dynamic tasks, by constraining activations to weighted groups of muscles, referred to as muscle synergies. Synergies are altered after neurological injury, such as stroke or cerebral palsy (CP), and may provide a method for improving subject-specific models of neuromuscular control. The aim of this study was to test whether constraining simulation to synergies could improve estimated muscle activations compared to EMG data. We evaluated modeled muscle activations during gait for six typically developing (TD) children and six children with CP. Muscle activations were estimated with: (1) static optimization (SO), minimizing muscle activations squared, and (2) synergy SO (SynSO), minimizing synergy activations squared using the weights identified from EMG data for two to five synergies. While SynSO caused changes in estimated activations compared to SO, the correlation to EMG data was not higher in SynSO than SO for either TD or CP groups. The correlations to EMG were higher in CP than TD for both SO (CP: 0.48, TD: 0.36) and SynSO (CP: 0.46, TD: 0.26 for five synergies). Constraining activations to SynSO caused the simulated muscle stress to increase compared to SO for all individuals, causing a 157% increase with two synergies. These results suggest that constraining simulated activations in inverse dynamic simulations to subject-specific synergies alone may not improve estimation of muscle activations during gait for generic musculoskeletal models.

## Introduction

Muscle synergies have been used as a method to describe how muscles are commonly activated during tasks such as walking, by identifying a low dimensional space of weighted muscle groupings (Bizzi and Cheung, 2013). These weighted groups of muscles have been shown to be altered among individuals with neurologic injuries, such as stroke or cerebral palsy (CP) (Cheung et al., 2009; Clark et al., 2010; Steele et al., 2015a; Tang et al., 2015; Shuman et al., 2016). Synergies appear to mature in stable patterns early in an individual’s lifespan, making them a potential platform for quantifying and modeling an individual’s motor control. In unimpaired children, synergies have been shown to be similar to adults after five years of age (Dominici et al., 2011; Rozumalski et al., 2017). For children with CP, synergies are altered but do not change over time, even after extensive surgical interventions with inpatient rehabilitation (Shuman et al., 2019). Although calculation of synergies has been used to describe muscle activation patterns in experimental data, these patterns have only begun to be applied to support musculoskeletal modeling. Using an individual’s synergies calculated from experimental data to inform neuromusculoskeletal simulations may improve estimates of an individual’s muscle coordination or response to interventions like assistive devices or rehabilitation.

Estimating muscle forces and activations are important for many questions asked with musculoskeletal modeling (Hicks et al., 2015). Examples include contributions of specific muscles to gait (Correa et al., 2011; Mcgowan et al., 2011; Steele et al., 2013; Mansouri et al., 2016) loads acting upon joints (Steele et al., 2012; Walter et al., 2014; Wesseling et al., 2015; Serrancolí et al., 2016), impacts of surgical interventions (Delp et al., 1996; Reinbolt et al., 2008; Fox et al., 2009), and use of orthotic devices (Hegarty et al., 2017; Rosenberg and Steele, 2017). However, when muscle activations are calculated using optimization-based methods, there are large variations in estimated muscle activations across studies (Tinler et al., 2018). Comparisons of modeled muscle activations to experimental data from electromyographic (EMG) recordings are frequently performed only qualitatively, broadly assessing timing and amplitudes (Hamner et al., 2010; Dorn et al., 2012; Hicks et al., 2015; Lerner et al., 2015; Wesseling et al., 2015; Krogt et al., 2016; Żuk et al., 2018b). Prior quantitative assessments revealed only moderate correlations between experimental and modeled muscle activations for both typically developing (TD) individuals and individuals with neurologic injuries (Heintz and Gutierrez-Farewik, 2007; Blazkiewicz, 2013; Żuk et al., 2018b; Veerkamp et al., 2019). A recent study found similar correlations between individuals with CP and TD individuals (Veerkamp et al., 2019). Prior research often used custom constraints, specifying when a muscle must be on and off, or other strategies to try to get better agreement between simulated activations and experimental measures from EMG data (Liu et al., 2008; Steele et al., 2012). Synergies may improve estimates of computed muscle activations by providing an alternate method to constrain which muscles are simultaneously activated based upon an individual’s EMG data (Ting et al., 2012).

Static optimization (SO) is a common algorithm used to estimate muscle activity that minimizes an objective function, such as minimizing the sum of squared muscle activations, while satisfying the system’s equations of motion. These optimization methods are theorized to reflect the strategies that unimpaired adults use to coordinate muscle activity. However, a recent study by Simpson et al. (2016) found that individual muscle activations could be adjusted to almost any level at any point in the gait cycle while still satisfying kinematic and kinetic constraints, suggesting that shapes of modeled activation patterns are driven predominantly by the choice of optimization function, rather than being required by the joint torques (Simpson et al., 2016). As high levels of co-contraction are a hallmark of gait in clinical populations like CP (Gage et al., 2009; Steele et al., 2017), other optimization criteria may be more appropriate when modeling pathologic gait (Steele et al., 2013; Sartori et al., 2017). If synergies reflect an individual’s neuromuscular control strategy, constraining to individualized synergy structures may help capture subject-specific activations patterns.

Synergies have previously been used to constrain muscle activity for musculoskeletal simulations, most prominently in forward dynamic simulations (Neptune et al., 2009; Mcgowan et al., 2011; Allen and Neptune, 2012; Sartori et al., 2013; Gopalakrishnan et al., 2014; Gonzalez-vargas et al., 2015; Mehrabi et al., 2019). Two studies have employed synergies with musculoskeletal modeling in pathologic gait of adult stroke survivors (Allen et al., 2013; Meyer et al., 2016). Most of these simulation studies have focused on tracking ideal synergy activation patterns as part of the optimization (Allen et al., 2013; Sartori et al., 2013; Gonzalez-vargas et al., 2015; Meyer et al., 2016; Serrancolí et al., 2016), which has allowed for performance similar to EMG tracking simulations, while reducing the number of input parameters. These methods require extensive model calibration achieved by adjusting model parameters like muscle activation delays, EMG scale factors, and tendon slack lengths such that the models closely match experimental kinematics and kinetics. These procedures are time and computationally expensive. One study applied synergy controls without EMG tracking and found better calculation of joint loads than individual EMG alone; however, this model was also highly calibrated for a single individual (Walter et al., 2014). Another study in the upper limb used optimization of synergy activations to model muscle activations during three-dimensional force generation and found that synergies better represented EMG data than independent muscle optimization (Borzelli et al., 2013).

The goal of this research was to evaluate whether constraining simulated muscle activations to an individual’s synergies calculated from experimental EMG data can improve estimates of muscle coordination for both TD children and children with CP. We hypothesized that the similarity between EMG data and activations for traditional SO methods would be lower for children with CP than TD peers, due to altered motor control. By specifying and constraining muscle activations to an individual’s synergies, we hypothesized that the similarity between EMG data and modeled activations would improve for both groups. This investigation examines whether synergy-based constraints alone, without changes to the model properties, can be used to improve fidelity of neuromusculoskeletal models to inform clinical or rehabilitation applications.

## Materials And Methods

### Participants

We retrospectively analyzed clinical motion analysis data collected at UZ Pellenberg, Belgium, for six children with CP (four males, age = 10.0 ± 3.3 years, mass = 33.2 ± 13.6 kg, height = 1366 ± 233 mm) and six TD children (three males, age = 8.9 ± 1.1 years, mass = 29.2 ± 1.8 kg, height = 1334 ± 39 mm). All children with CP were in Gross Motor Function Classification System (GMFCS) Levels I or II. Marker trajectories were tracked using a 10–15 camera VICON system (Nexus 1.8.4. Vicon-UK, Oxford, United Kingdom) sampled at 100 Hz. Each trial consisted of barefoot walking at a self-selected speed on a 10 m walkway. Ground reaction forces were collected using two AMTI force plates sampled at either 1000 or 1500 Hz. The number of over ground walking trials ranged between 4 and 8 for CP and 3 and 10 for TD.

### Electromyography

Surface EMG data (Wave Wireless EMG, Cometa, Bareggio, Italy) were collected at either 1000 or 1500 Hz from eight muscles bilaterally (gluteus medius, rectus femoris, vastus lateralis, medial hamstrings, lateral hamstrings, tibialis anterior, gastrocnemius, and soleus) during clinical gait analysis. Because we were using retrospective clinical data, not all muscles were recorded for every trial and, for some individuals, a single muscle was missing from all trials (right vastus lateralis in CP03, CP04, and CP05, left tibialis anterior in TD02, and left rectus femoris in TD04). Raw EMG data were bandpass filtered between 20 and 500 Hz upon collection. We calculated a linear envelope for each muscle by high-pass filtering at 20 Hz, rectifying the data, and low-pass filtering at 6 Hz (Shuman et al., 2017). Prior to calculating synergies, we concatenated the middle 80% of EMG data for all available trials for each participant to maximize the amount of data for synergy analysis while removing periods of transient acceleration or deceleration near the beginning and end of each trial (Oliveira et al., 2014; Shuman et al., 2017). Each trial contained three to five strides of EMG data. The concatenated data were down-sampled to 100 Hz and scaled to a peak amplitude of one for each muscle.

### Synergy Analysis

For each individual, we calculated synergies with weighted non-negative matrix factorization (WNMF) using the Matrix Factorization Toolbox (Kim and Park, 2007; Li and Ngom, 2013) in Matlab (MathWorks, Inc., Natick, MA, United States) from the concatenated EMG data. We have previously used WMNF to accommodate clinical EMG data with poor or missing channels by assigning a weight of zero to those data points, allowing us to maximize data for synergy analysis (Shuman et al., 2018, 2019). Aside from the missing EMG channels noted above, all muscles were recorded in at least two trials within the concatenated session. WNMF numerically identifies a set of synergy weights (*W*_mxn_) which are activated (*C*_nxt_) such that the processed *EMG*_mxt_ data are approximated, where *m* is the number of muscles (7 or 8), *n* is the number of synergies (2–5), and *t* is the number of time points in the concatenated EMG session:

EMG=W⁢x⁢C+error

**FIGURE 1:**
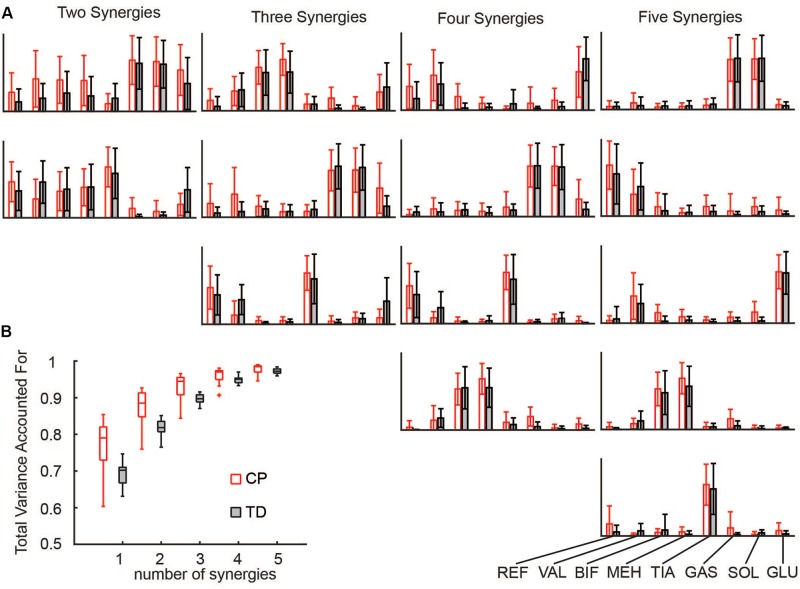
Synergies calculated from EMG data: **(A)** Muscle weights for two to five synergies for CP and TD. **(B)** The total variance in EMG data accounted for by a given number of synergies was greater for children with CP than TD peers. The “+” represents outlier points (greater than the 75th percentile + 1.5^∗^IQR or less than the 25th percentile – 1.5^∗^IQR.

Synergies were calculated for each side (unilaterally) using the following WNMF settings: 50 replicates, 1000 maximum iterations, 1 × 10^–4^ minimum threshold for convergence, and 1 × 10^–6^ threshold for completion. Synergy weights and activations were scaled such that the maximum weight in a synergy was one. Similar to prior research, reconstruction of the EMG data by *n* synergies accounted for more EMG variance in CP than to TD for all numbers of synergies (Figure 1) (Steele et al., 2015a).

### Musculoskeletal Modeling

We used marker trajectories from an extended marker set, based upon the Plug-in-Gait (PiG) model, to scale a generic 19 degree-of-freedom and 92 musculotendon actuator model in OpenSim version 3.2 (Delp et al., 1990, 2007; Anderson and Pandy, 1999). We used inverse kinematics to calculate joint angles by minimizing the error between the experimental markers and virtual model markers. The average RMS marker error was 0.92 ± 0.19 cm and the average maximum marker error was 2.64 ± 0.97 cm (Hicks et al., 2015). The force plate data used in this study had a threshold applied upon collection where forces under 25 N were not recorded. Thus, to avoid dynamic inconsistencies in our models occurring near heel strike and toe off, we limited our investigation to single-limb stance. We performed a residual reduction analysis to improve dynamic consistency in our models by making small adjustments to joint angles and adjusting the position of the center of mass in the lumped head, arms, trunk, segment (Thelen and Anderson, 2006). A total of 88 simulations of single-limb stance phase (44 CP, 44 TD) were generated. Swing phase was evaluated for each limb when the opposite limb was in stance. The number of simulations per individual ranged between 3–13 for CP and 5–10 for TD.

**FIGURE 2:**
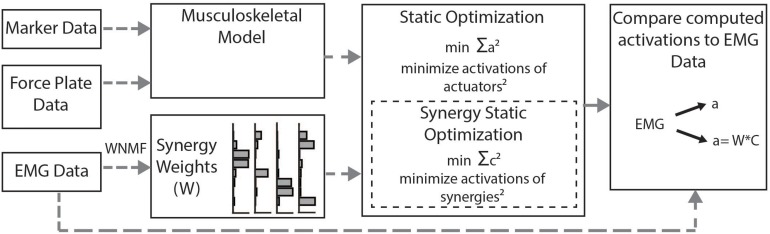
Musculoskeletal modeling framework: Musculotendon activations are computed from the musculoskeletal model using the static optimization algorithm or the synergy static optimization algorithm and compared to measured EMG data. Static optimization minimizes the sum of all 92 actuators squared. Synergy static optimization groups muscles together using synergy weights from the measured EMG data and minimizes the sum of the activations of those synergies squared.

We calculated simulated muscle activations using two methods (Figure 2). First, we used the standard SO algorithm in OpenSim (Anderson and Pandy, 2001). SO estimates muscle forces that satisfy joint inverse dynamics at each point in time while accounting for muscle force-length properties. The cost function employed by SO minimizes muscle stress as the sum of squared muscle activations (Crowninshield and Brand, 1981; Kaufman et al., 1991; Anderson and Pandy, 2001). To evaluate whether constraining to synergies improved estimates of muscle activity, we used the synergy optimization (synSO) plug-in previously described by Steele et al. (2015b). SynSO allows the user to specify weighted groups of muscles to be commonly activated while minimizing the sum of squared synergy activations. For each synergy, the synergy weights we calculated from experimental EMG data were applied to the corresponding musculotendon actuators for each muscle. Note that the synergy weights were calculated from concatenated EMG data representing all parts of the gait cycle (not just single-limb stance). Thus, synergy weights for the gastrocnemius were applied to both the medial and lateral gastrocnemius actuators, weights for the medial hamstrings were applied to both semimembranosus and semitendinosus actuators, weights for the lateral hamstrings were applied to both biceps femoris long head and short head actuators, and weights for the rectus femoris, vastus lateralis, tibialis anterior, and soleus were all applied to their individual musculotendon actuators. As only 26 of the model’s 92 musculotendon actuators were accounted for with EMG data, the remaining 66 musculotendon actuators were independently activated as in SO. For each trial, we evaluated SynSO for sets of two, three, four, or five synergies for each participant. Synergies were calculated and applied independently for each individual and each leg (e.g., in a four-synergy simulation, we calculated and used four synergies for the right leg and four synergies for the left leg). Constraining activations through SynSO to fewer synergies reduced the number of successful simulations. For all numbers of synergies, more simulations were successful in CP than TD (e.g., 95 vs 86% for five synergies; 45 vs 34% for two synergies).

### Outcome Measures

To determine whether constraining to synergies resulted in simulated muscle activations that were more similar to measured EMG data, we calculated the cosine similarity between the filtered EMG data and simulated muscle activations for SO and SynSO. We determined that the similarity due to chance was 0.55, which we calculated as the average cosine similarity across all individuals and EMG channels to 1000 random vectors with a truncated Laplacian distribution (Tresch et al., 2006). Thus, cosine similarity was normalized such that the similarity due to chance was given a value of zero (e.g., a similarity of 0.55 would have a normalized similarity of 0.0). We examined the similarity for each muscle by concatenating the simulated activations from all trials and calculating the cosine similarity to the corresponding measured EMG data. For muscles modeled with multiple musculotendon actuators, we averaged activations for comparison to EMG. We calculated the average similarity for each participant across all muscles. We compared the average normalized similarity of estimated activations to EMG data between SO and SynSO, and between single-limb stance and swing for each algorithm. We also computed the change in summed muscle stress (overall and by muscle) as the summed activation of each muscle and computed peak activation of muscles. Descriptive statistics (median and IQR) were used to compare normalized similarity, muscle stress, and peak simulated activations.

## Results

**FIGURE 3:**
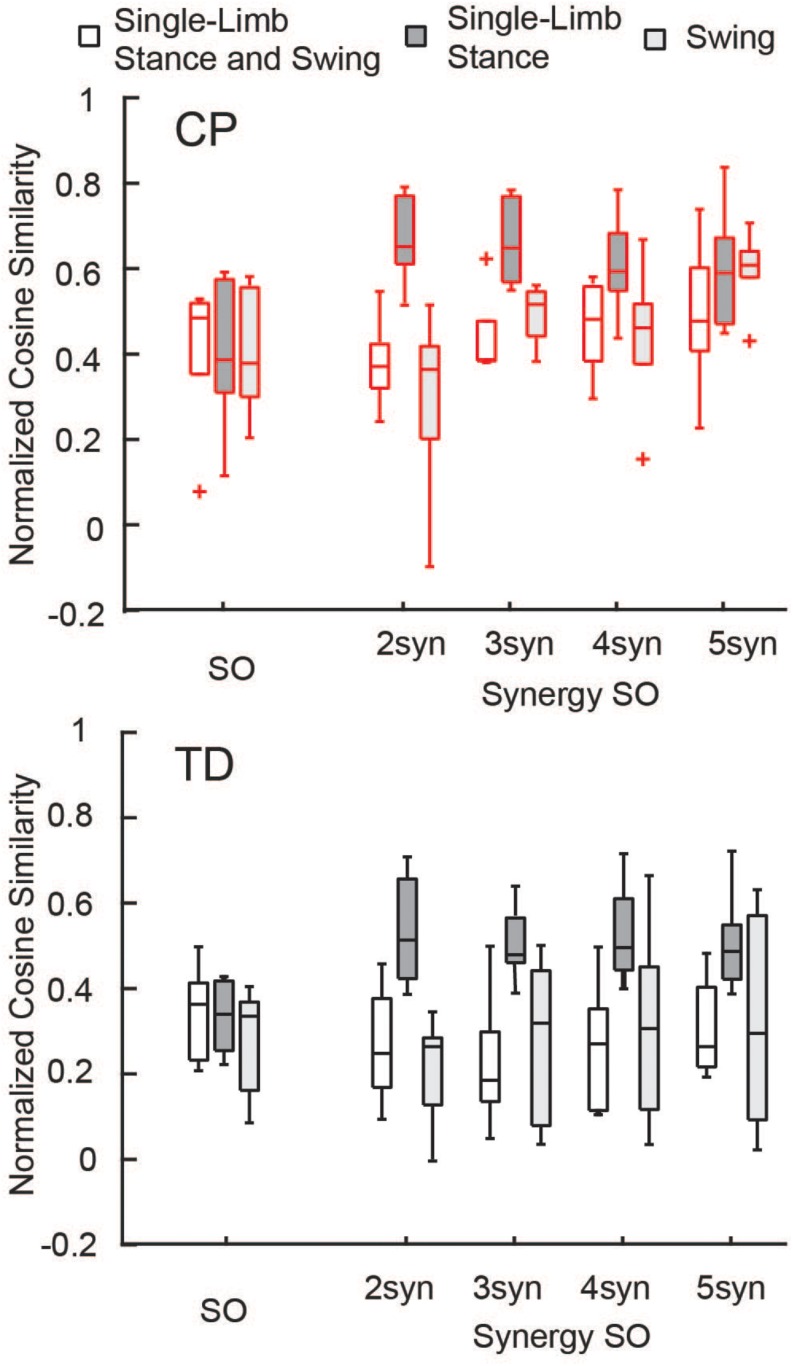
Normalized similarity of simulated activations and EMG data: Cosine similarity was used to examine how well estimated activations from simulation represented experimental EMG data for the TD and CP groups during single-limb stance and/or swing. Similarity was normalized such that zero equals similarity due to random chance and one equals perfectly similarity. Median similarity was better than chance for both CP and TD for SO and SynSO. Single-limb stance phase was better represented by SynSO than SO. The “+” represents outlier points (greater than the 75th percentile + 1.5^∗^IQR or less than the 25th percentile – 1.5^∗^IQR.

The similarity of estimated activations and experimental EMG data was similar between SO and SynSO, but generally poor for both algorithms. The normalized similarity across single-limb stance and swing between EMG and simulated muscle activations from SO was higher in CP [median (IQR): 0.48 (0.17)] than in TD [0.36 (0.18)] (Figure 3). Normalized similarity for SynSO in CP was less than SO when fewer synergies were used, with values of 0.37 (0.10), 0.38 (0.09), 0.48 (0.17), and 0.47 (0.19) for two to five synergies, respectively. In TD, similarity from SynSO was less than in CP and was lower than SO with a similarity of 0.24 (0.21), 0.19 (0.16), 0.27 (0.24), 0.26 (0.19) for two to five synergies. For SynSO, estimates of muscle activity were more similar to EMG data during single-limb stance phase (CP: 0.59–0.65, TD: 0.48–0.51) than swing (CP: 0.36–0.61, TD: 0.26–0.32). For SO, there was no difference in single-limb stance [CP: 0.39 (0.26), TD: 0.34 (0.16)] or swing [CP: 0.38 (0.26), TD: 0.33 (0.21)] for estimates of muscle activity compared to EMG data.

**FIGURE 4:**
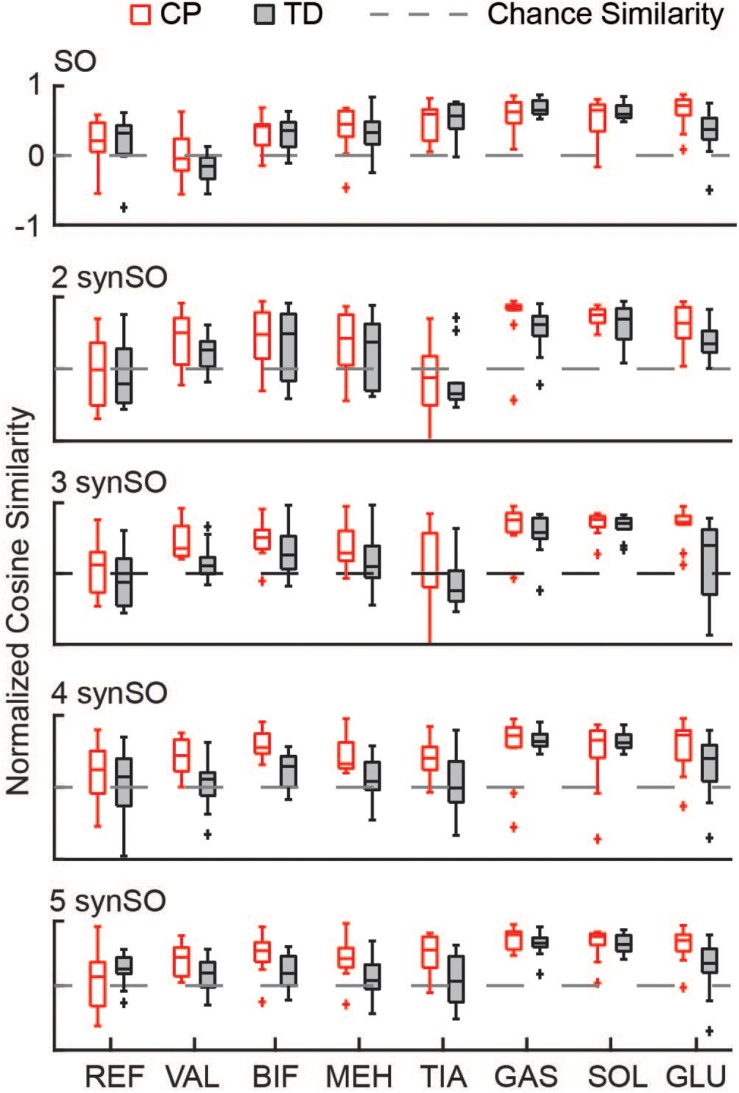
Similarity of individual muscles: The similarity of each muscle was compared for SO and SynSO for CP and TD groups. Similarity was normalized such that zero equals similarity due to random chance and one equals perfectly similarity. Activations computed with SO had the lowest similarity to EMG data for the rectus femoris (REF), vastus lateralis (VAL), and biceps femoris (BIF). Activations computed with SynSO had poor similarity to EMG data for the tibialis anterior (TIA), REF, and BIF (for the TD group). The gastrocnemius (GAS) and soleus (SOL) had the greatest similarity to EMG data for both algorithms. The “+” represents outlier points (greater than the 75th percentile + 1.5^∗^IQR or less than the 25th percentile – 1.5^∗^IQR.

**FIGURE 5:**
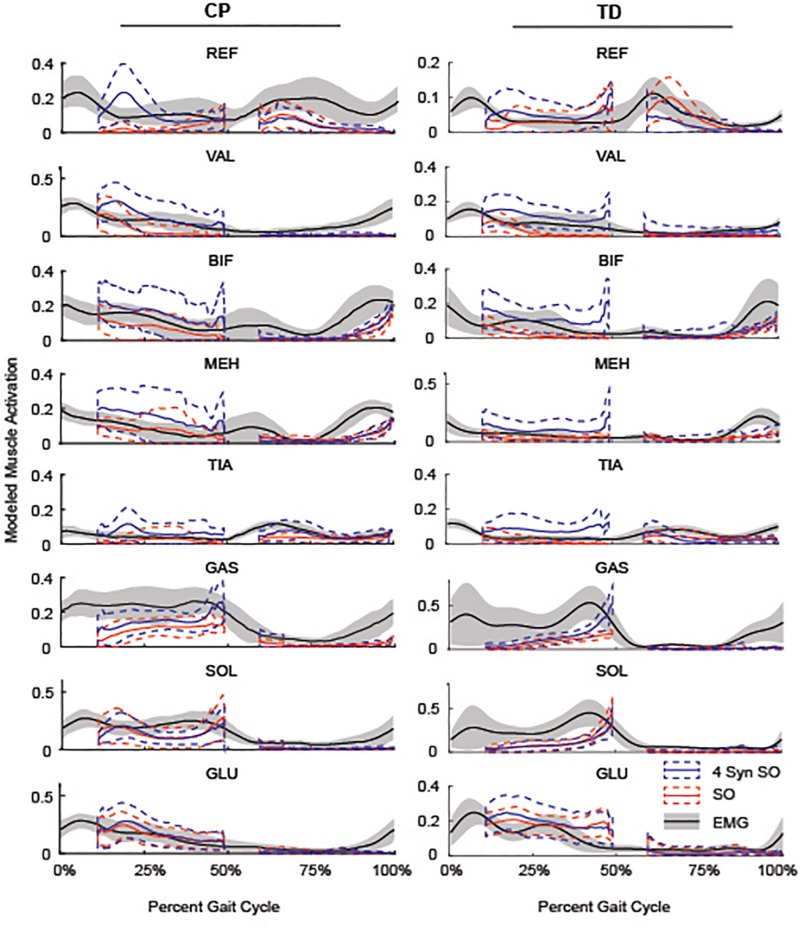
Average SO, 4 SynSO, and EMG activation patterns for CP and TD: The modeled activation tended to be higher for SynSO than SO for most muscles in both TD and CP during single-limb stance. EMG activations are scaled to the maximum activation in either SynSO or SO.

Similarity to EMG data was highly variable between individuals. The gastrocnemius, soleus, and tibialis anterior were the most similar muscles to EMG recordings using SO (Figure 4). SynSO tended to improve the similarity to EMG for the plantar flexors (CP: median soleus similarity increased from 0.65 for SO to 0.76 for three SynSO) and decreased the similarity to EMG for the tibialis anterior (TD: median similarity decreased from 0.57 for SO to –0.24 for three SynSO). The similarity of the gluteus medius to EMG was higher in CP [SO: 0.72 (0.22)] than TD [0.37 (0.30)] but did not become more similar when SynSO was employed. The hamstrings and rectus femoris tended to become less similar to EMG when constrained to SynSO for the TD group and had only small changes in CP. The vastus lateralis tended to be only as similar as chance using SO and became more similar to EMG for all numbers of synergies in CP, but was still poorly represented in the TD group (SO: –0.16; five SynSO 0.19). Examination of the activation patterns between SO and SynSO showed that most muscles were recruited to higher amplitudes in single-limb stance (Figure 5).

**FIGURE 6:**
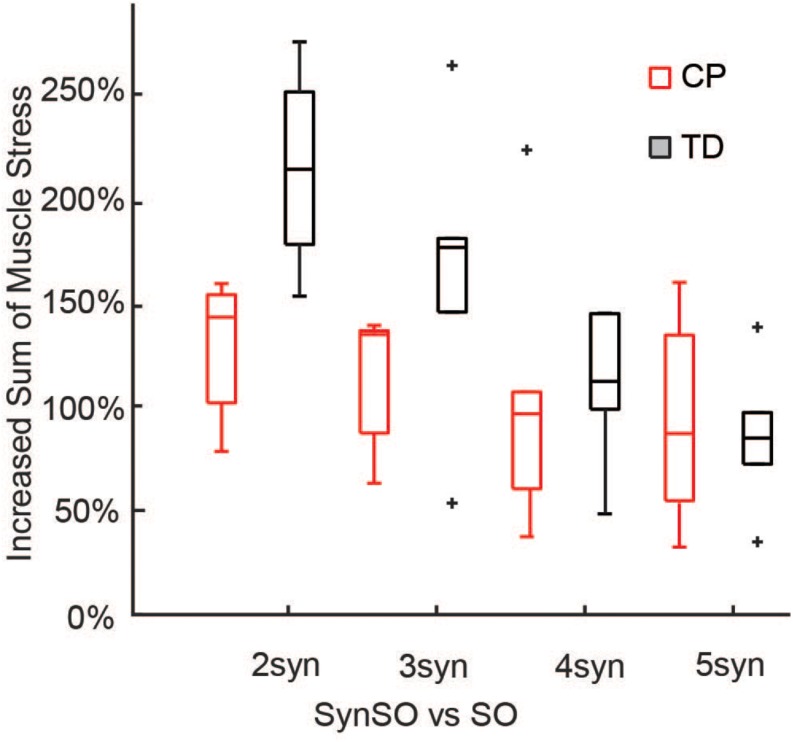
Increased sum of muscle stresses for SynSO: Muscle stress measured as the muscle activations squared increased for both TD and CP across all number of synergies. Increases in muscle stress were highest for two synergies and lowest for five synergies. The “+” represents outlier points (greater than the 75th percentile + 1.5^∗^IQR or less than the 25th percentile – 1.5^∗^IQR.

Simulated muscle stress increased in SynSO relative to SO for all individuals and was highest for the two synergy solutions (Figure 6) with an increase of 157% (72%). Simulated muscle stress is a rough estimate of energetic cost, indicating that the constraints of SynSO find solutions requiring greater effort. Muscles that were constrained to a synergy increased muscle stress by 72% (61%) for two synergies and 64% (45%) for five synergies compared to SO. Muscles that were not constrained to a synergy in SynSO increased muscle stress by 323% (235%) for two synergies and 147% (98%) for five, suggesting that constraining to synergies for muscles with EMG data led to greater dependence on non-constrained musculotendon actuators in the model. Despite the large changes in summed muscle stress, changes in peak activation were less than 10% for over 60% of the muscles across all numbers of synergies in SynSO.

**FIGURE 7:**
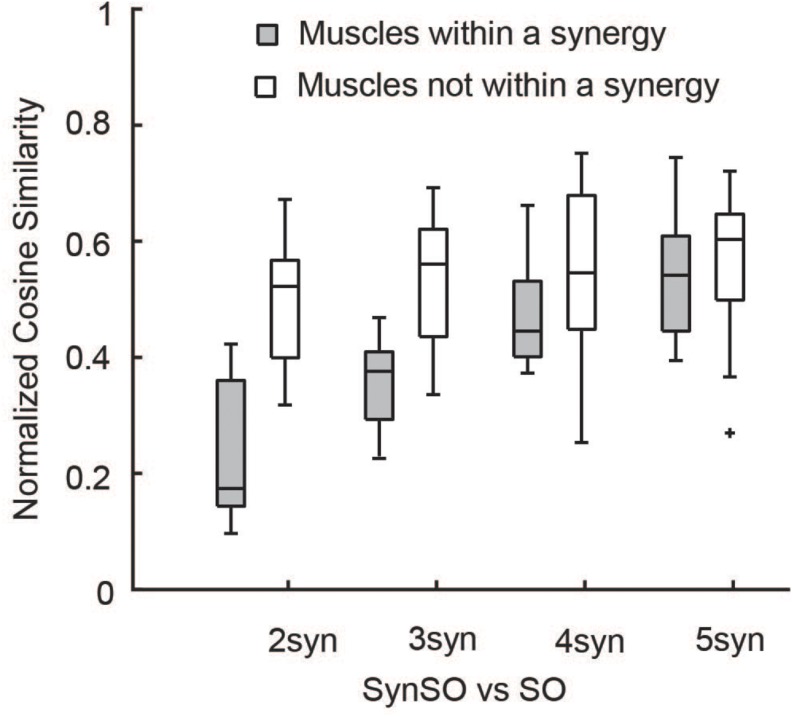
Similarity of activations computed with SO and SynSO: Activations computed with SynSO were different than those calculated from SO, for both muscles that were constrained to a synergy and muscles that did not have EMG data and were independently activated. Similarity was normalized such that zero equals similarity due to random chance and one equals perfectly similarity. The “+” represents outlier points (greater than the 75th percentile + 1.5^∗^IQR or less than the 25th percentile – 1.5^∗^IQR.

SynSO caused changes in simulated activations both for muscles included within synergies and muscles that were independently controlled (no EMG data, Figure 7). Muscles that were constrained to synergies demonstrated the largest changes, with a normalized similarity between SO activations and SynSO of 0.17 (0.22) for two synergies and 0.54 (0.16) for five synergies. Muscles that were independently controlled had smaller changes, with a normalized similarity between SO and SynSO activations of 0.52 (0.09) for two synergies and 0.60 (0.14) for five synergies.

## Discussion

We investigated whether constraining musculoskeletal simulations to an individual’s synergies calculated from experimental EMG data could improve estimations of muscle activation from inverse dynamic simulations of gait. Across all subjects, SynSO caused changes in estimated muscle activation patterns compared to traditional SO. Compared to SO, estimated muscle activations using SynSO tended to better match EMG data during single-limb stance for both TD and CP individuals. However, SynSO also tended to estimate activations that were less similar to EMG data during swing, such that overall SynSO did not better estimate EMG data than SO. These differences may indicate that synergies better represent coordination patterns in stance than swing. For both algorithms, the similarity to experimental EMG data for both CP and TD groups was generally poor, emphasizing the need for new methods to model muscle activity for analyses of human movement. As modeling methods are used to inform rehabilitation or assistive device design, identifying the changes in modeling and simulation methods required to accurately capture muscle coordination will be critical to ensure that predicted effects will be relevant for a specific individual.

The correlations found in this study between EMG and SO were similar to those previously reported (Heintz and Gutierrez-Farewik, 2007; Blazkiewicz, 2013; Żuk et al., 2018b; Veerkamp et al., 2019). These four studies demonstrate variability both between individuals and across muscles, similar to our results. The best-represented muscles during gait were the plantar flexors, while the worst represented muscles were the knee extensors and hamstrings, consistent with our results for both CP and TD (Heintz and Gutierrez-Farewik, 2007; Blazkiewicz, 2013; Żuk et al., 2018b).

Selection of an appropriate number of synergies is challenging for this type of problem. To avoid biasing our results based upon and *ad hoc* threshold, we computed our results over a range of two to five synergies. We chose to apply a minimum of two synergies, as a representation of gross flexion and extension which has been previously been found to well represent data in infants and individuals with CP (Dominici et al., 2011; Steele et al., 2015a). As additional synergies were added, muscle activation patterns within each synergy became more independent (e.g., the tibialis anterior was largely independent in the five-synergy solution for CP and TD), more closely representing the conditions in SO (Figure 1). Constraining to a smaller number of synergies led to higher levels of overall muscle stress, indicating an overall less optimal solution. As SynSO did not tend to improve correlation with EMG data for any number of synergies, we were unable to find an optimal number of synergies needed for either group.

Our results using synergies to improve estimations of muscle activation during gait contrasts with previous studies (Borzelli et al., 2013; Walter et al., 2014; Meyer et al., 2016; Serrancolí et al., 2016) which found generally good estimation of EMG with synergies. The differences between the previous work and our results here broadly fit into three categories: the optimization criteria, the challenge of relating EMG amplitudes to neural excitations, and generic musculoskeletal properties. In the prior studies of Walter et al. (2014), Meyer et al. (2016), and Serrancolí et al. (2016), EMG shape tracking was used as part of the optimization algorithm. In this study, we sought to model muscle activations through a modified SO cost function which minimized synergy activations squared, consistent with the optimization previously implemented by McKay and Ting (2012) and Borzelli et al. (2013). This cost was motivated by the traditional physiologically motivated cost functions which seek to minimize fatigue or load in individual muscles (Crowninshield and Brand, 1981; Anderson and Pandy, 2001; Ackermann and Van Den Bogert, 2010), while constraining the space of allowable muscle activations to specified patterns of coactivation. The constrained search space resulted in higher muscle stresses than what is found with independent actuation, consistent with prior studies (McKay and Ting, 2012; Borzelli et al., 2013). We note that minimizing synergy activations squared alters the cost function, removing the direct physiological relationship to muscle stress. A prior investigation by McKay and Ting (2012) in cats suggests that minimizing synergy activations squared, as performed in this study, results in higher muscle stress than minimizing muscle stresses squared subject to synergy constraints. A further difference is that, unlike the prior studies (McKay and Ting, 2012; Borzelli et al., 2013), we did not have EMG data for all muscles in the model and thus allowed the unmonitored muscles to have their activations optimized independently.

The challenges in directly comparing EMG to musculoskeletal modeling have been well documented (Farina et al., 2004; Sartori et al., 2017) and include scaling EMG to peak neural excitations (Ting et al., 2012; Borzelli et al., 2013; Kristiansen et al., 2015), electromechanical delays (Durandau et al., 2018), as well as interpretation of EMG stemming from inter-step variability, crosstalk, cancelation, measurement orientation, and pre-processing decisions (Farina et al., 2004; Shuman et al., 2017). In this study, we modeled activations using subject-specific synergies whose weights were derived from EMG data normalized by peak measured EMG amplitude during walking. This choice was necessitated by our use of retrospective data and represents the simplest implementation of synergies into musculoskeletal modeling. To compensate for the uncertain scaling parameters between EMG and neural activation, previous forward dynamic simulations have tracked activation patterns but allowed the relative weights of the modeled muscles to vary either through a minimization of muscle stress with synergy activation tracking (Neptune et al., 2009; Allen and Neptune, 2012) or as part of the initial EMG tracking calibration process (Walter et al., 2014; Meyer et al., 2016; Serrancolí et al., 2016). Alternate methods of scaling synergies experimentally, such as by a maximum voluntary contraction or the use of force-to-EMG measurements (Borzelli et al., 2013), require the collection and integration of additional data, significantly complicating the implementation. Although the choice of amplitude scaling prior to calculating synergies can impact the relative weights of muscles within a given synergy (Shuman et al., 2017), a recent investigation by Kieliba et al. (2018) found nearly identical synergy structures between EMG data normalized by maximum voluntary contractions or peak activations in healthy adults. The consistency of these synergy structures suggests that the relative weights of muscles within a synergy scaled by experimental data may only have a small impact on our results.

A key limitation of our ability to model muscle activations using SynSO is the lack of any electromechanical delay, which neglects activation/deactivation dynamics. In those studies that used EMG shape tracking, the electromechanical delay was also uniquely scaled for each muscle (Walter et al., 2014; Meyer et al., 2016) or applied from the literature (Serrancolí et al., 2016). In a *post hoc* analysis, we evaluated the impacts of including a delay between EMG and modeled activations of 10–100 ms but found inconsistent impacts on similarity between phases of the gait cycle and number of synergies included in the optimization. For the previous studies which used an SO-based algorithm, the investigations were limited to examining muscle activity during a isometric force generation task across a variety of directions (McKay and Ting, 2012; Borzelli et al., 2013), negating the impact of activation dynamics. Conversely, for dynamic tasks such as walking, in which a gait cycle may take approximately one second, even a 50 ms delay may have substantial impacts on the similarity between simulated muscle activity and experimental EMG. This is likely a defining difference in the higher accuracy of estimated muscle activity in previous studies incorporating synergies compared to the results of this research.

A significant limitation of this study was the use of generic musculoskeletal models. While generic models have the advantage of minimizing the amount of data that must be collected for any individual, they achieve this by including sample-based assumptions about geometry (e.g., muscle attachment points, and bone geometry) and muscle properties (e.g., activation delays, maximum muscle forces, and tendon lengths). These assumptions can have large impacts on estimated muscle activations (Correa et al., 2011; Ackland et al., 2012; Serrancolí et al., 2016; Roelker et al., 2017; Sartori et al., 2017; Żuk et al., 2018a; Hegarty et al., 2019) and may not represent individual properties (Zajac, 1989), especially for individuals not well represented by the population used to develop the models, such as children or individuals with CP (Barber et al., 2012; Barrett and Barber, 2013; Mudge et al., 2014; Handsfield et al., 2016). We found variable results across participants and muscles in both TD and CP groups emphasizing the limitations of generic musculoskeletal models to capture heterogeneity in our populations. To address this, previous studies examining synergies in musculoskeletal modeling that use EMG shape tracking tune musculotendon properties as part of the model calibration (Walter et al., 2014; Meyer et al., 2016; Serrancolí et al., 2016), but these parameters are difficult to validate. Other musculoskeletal studies incorporate imaging data to personalize bone and muscle geometry (Barber et al., 2011; Scheys et al., 2011; Kohout et al., 2013; Handsfield et al., 2016; Modenese et al., 2016; Sartori et al., 2017). Incorporation of subject-specific geometry and muscle properties may influence the utility of synergies in modeling muscle activations, but the degree of personalization required remains unclear.

This study demonstrated that muscle activations estimated from SO using generic musculoskeletal modeling does not accurately predict EMG profiles for children with CP or TD peers. Constraining activation patterns to experimentally measured synergies increased estimated muscle stresses, but did not improve the estimation of muscle activations for either group. These findings suggest that when generic musculoskeletal models are used, constraining muscle activations to synergistic patterns alone may not improve estimation of muscle activations during gait. Additional methods, such as tuning of muscle-tendon model parameters, may be required to create neuromusculoskeletal simulations that can accurately represent muscle coordination for rehabilitation or assistive device applications.

## Data Availability Statement

The datasets generated for this study and source code for the plug-in (Steele et al., 2015b) are available on request to the corresponding author.

## Ethics Statement

The studies involving human participants were reviewed and approved by Commissie Medische Ethiek (KU Leuven). Written informed consent from the participants’ legal guardian/next of kin was not required to participate in this study in accordance with the national legislation and the institutional requirements.

## Author Contributions

BS, KD, MS, and KS conceptualized the study. BS analyzed the data. BS, MG, KD, MS, and KS interpreted the data, and drafted and revised the manuscript. All authors read and approved the final manuscript.

## Conflict of Interest

The authors declare that the research was conducted in the absence of any commercial or financial relationships that could be construed as a potential conflict of interest.
